# Elevated white blood cell count, decreased hematocrit and presence of macrohematuria correlate with abdominal organ injury in pediatric blunt trauma patients: a retrospective study

**DOI:** 10.1186/s13017-015-0034-5

**Published:** 2015-09-15

**Authors:** Yehuda Hershkovitz, Sergei Naveh, Boris Kessel, Zahar Shapira, Ariel Halevy, Igor Jeroukhimov

**Affiliations:** Division of Surgery, Assaf Harofeh Medical Center, Zerifin 70300, affiliated to the Sackler Faculty of Medicine, Tel Aviv University, Tel Aviv, Israel; Trauma Unit, Hillel Yaffe Medical Center, Hadera, 38100 Israel

**Keywords:** Abdominal trauma, Pediatric trauma management, CT in blunt trauma

## Abstract

**Introduction:**

Computerized tomography (CT) has become an important diagnostic modality in trauma patients. Pediatric patients are particularly susceptible to ionized radiation making liberal CT use in this age group unacceptable. We aimed to identify parameters that might predict abnormal findings on abdominal CT leading to patient management changes.

**Methods:**

Data on blunt trauma patients up to 15 years of age admitted to Assaf Harofeh Medical Center from January 2007 to October 2014 was retrospectively collected. All patients with abdominal CT scan as part of initial assessment were included. Medical and surgical data were extracted from the medial charts. Patients were divided into two groups. Group I: patients whose management was changed solely based on abdominal CT findings and Group II: patients with normal abdominal CT. The groups were compared by all the data parameters.

**Results:**

Overall, 182 patients were included in the study. The groups were comparable by age and mechanism of injury. Management changes based on CT findings were found in 68 (37.4 %) patients. White blood cell count >14000, abnormally low hematocrit level and macrohematuria were associated with a diagnosis of intra-abdominal injury requiring patient management changes (*p* < 0.05). Group I patients had longer LOS. Fifteen patients (22 %) required active intervention based solely on CT findings. Physical examination, arterial blood gases and initial radiology examinations results did not correlate with abdominal CT findings.

**Conclusions:**

Elevated WBC, decreased hematocrit and presence of macrohematuria strongly correlate with abdominal CT findings and lead to changes in patient management.

## Introduction

The potential risk of radiation damage caused by CT scan dictates careful use of this imaging modality [1]. While in the management of a stable adult patient with blunt abdominal trauma the indications for a CT scan are established [2], its use in the pediatric population is still a matter of debate. It has been shown that the mechanism of injury as a sole indication for obtaining a CT scan in pediatric trauma victims does not clearly predict significant findings affecting patient management [3, 4].

The indications for abdominal imaging after blunt trauma are usually based on physical examination and mechanism of injury suggestive of intra-abdominal injury [5]. However, the clinical evaluation of children with potential abdominal trauma may be limited and often unreliable, especially in multisystem injuries [6].

Although abdominal CT is considered the best imaging modality for diagnosing intra-abdominal injury (IAI), less than 15 % of pediatric patients sustaining blunt trauma are found to have IAI on CT [5].

Increasing awareness of the hazards that exposure to radiation carries, especially in pediatric population, and given the scarcity of traumatic CT findings leading to intervention in these patients, led us to clarify factors that may help in the use of CT on a more selective basis.

The purpose of this study was to define objective clinical parameters on initial evaluation of children with blunt abdominal trauma that predict traumatic findings on abdominal CT scan which would influence the management plan.

## Methods

A retrospective observational cross sectional study was performed at Assaf Harofeh Medical Center. The study was approved by the Institutional Review Board.

Charts of pediatric blunt trauma patients admitted from January 1, 2007 to September 30, 2014 that underwent abdominal CT as a part of initial evaluation were reviewed. All blunt trauma patients up to 15 years old with Abbreviated Injury Scale (AIS) severity score indicating abdominal injury were eligible for the study. Patients with decreased level of consciousness (GCS < 12) and patients who did not undergo abdominal CT were not included. Data regarding patient demographics, mechanism of injury, initial physical examination findings, Glasgow Coma Scale (GCS) on admission, laboratory data, radiologic interpretation of chest and pelvic roentgenograms, focused assessment with sonography in trauma (FAST) results, CT scan findings and required surgical procedures and length of hospital stay (LOS) were extracted from the medical charts.

Our institutional policy dictates overnight observation for every pediatric patient who has a CT scan as a part of trauma management.

Change in patient management was defined as an alteration in management based directly on the CT findings.

An abnormal CT was defined as a scan exhibiting any traumatic abnormality either apparent or occult (finding of free fluid in the peritoneal cavity).

The patients were divided into two groups. Group I included patients whose management was changed due to abdominal CT findings. This group included either patients admitted to the Intensive Care Unit (ICU) or patients hospitalized longer than 24 h as a result of the findings on abdominal CT. All the patients undergoing acute interventions including laparotomy, angiography/embolization or ERCP necessarily fell into this Group. Group II included patients whose management was not affected by abdominal CT findings.

Findings on physical examination were classified as normal or abnormal. Abdominal examination was considered abnormal if abrasions, contusions, seat-belt sign, abdominal distension, or tenderness on palpation were noted. Positive findings on the examination of extremities included presence of abrasions, lacerations, deformity/fracture, or tenderness on palpation. Laboratory tests obtained only on arrival were included. Laboratory results were classified as normal and abnormal (according to the patient's age). White blood cell (WBC) count up to 11,000 was considered normal, mildly elevated ranging from 11000 to 14000 and markedly elevated above 14000. Chest and pelvis roentgenogram were screened for trauma-related pathology. FAST was considered positive if intraperitoneal fluid was demonstrated.

### Statistical analysis

In order to compare quantitative (continuous) variables between the two independent groups, the two sample *t*-test was applied as well as the non-parametric Mann–Whitney test. The association between the two categorical variables was assessed using either the Chi-square test or Fisher's exact test. All statistical tests applied were two-tailed. A p value less than 0.05 was considered statistically significant. Statistical analysis was performed using the Statistical Package for the Social Sciences (SPSS) software.

## Results

Overall, 397 pediatric patients with abdominal trauma were admitted to our Medical Center during the study period. One hundred and eighty-two patients entered the study. The rest of the patients either had GCS lower than 12 (40 patients) or did not undergo abdominal CT (175 patients).

The mechanism of injury, demographics, GCS and Injury Severity Score (ISS) are summarized in Table 1. There was no difference in the patient characteristics between the two groups, with the exception of the ISS which was higher in Group I.

**Table 1:** Patient characteristics

		Group I 68 patients	Group II 114 patients	*P* value
Age		9.1 ± 4.1	8.7 ± 3.9	>0.05
Gender (male / female)		54 / 14	81 / 33	>0.05
Mechanism of injury	PHBC**	20.6 %	30.7 %	>0.05
	MVA passenger***	5.9 %	10.5 %	
	Fall	20.6 %	18.4 %	
	Assault	20.6 %	18.4 %	
	Bike	27.9 %	19.3 %	
	BHC****	4.4 %	2.6 %	
ISS*	1–8	13.2 %	78.9 %	<0.05
	9–14	17.6 %	11.4 %	
	15–24	51.5 %	4.4 %	
	>25	17.6 %	5.3 %	

*ISS, Injury Severity Score; ** PHBC; Pedestrian hit by car. *** MVA – Motor Vehicle Accident. **** BHC – Bicycle Hit by Car

*ISS, ** PHBC, *** MVA, **** BHC. *ISS*, Injury Severity Score; *PHBC*; Pedestrian hit by car; *MVA*, Motor vehicle accident; *BHC*, Bicycle hit by car

A positive physical examination did not predict clinically relevant findings on CT (Table 2). This relates to the analysis of positive findings on physical examination irrespective of body region.

**Table 2:** Physical examination findings

	Group I	Group II	*p*
Head	33.8 %	25.4 %	>0.05
Neck	1.5 %	4.4 %	>0.05
Chest	14.7 %	9.6 %	>0.05
Abdomen	29.4 %	41.2 %	>0.05
Back	10.3 %	12.3 %	>0.05
Upper limbs	19.1 %	14.9 %	>0.05
Lower limbs	22.1 %	34.2 %	>0.05

CT revealed abdominal organs injury in 68 (37.4 %) patients (Table 3). Solid organs were injured in most cases (spleen 31, liver 19, kidney 17). Management altered by CT scan involved the need for active intervention in 15 out of 68 patients (22.1 %) [10 (14.7 %) patients underwent laparotomy, 2 (2.9 %) required an angiographic arterial embolization (for splenic and liver injury) and 3 patients (4.4 %) had an ERCP for suspected pancreatic duct injury]. Observation only sufficed in all other patients. Three out of 10 laparotomies (30 %) were performed in patients whose only finding on CT was free peritoneal fluid. Small bowel laceration was found in 2 cases and 1 had a mesenteric tear.

**Table 3:** Group I CT findings

Injured organ	Number	Percent
Solid organ injury (liver, kidney or spleen)	52	76.4 %
Pancreas	7	10.3 %
Retroperitoneal hematoma	3	4.4 %
Moderate amount of free fluid	3	4.4 %
Bowel	2	2.9 %
Urinary bladder	1	1.5 %

Overall LOS for Group I patients was 7.3 ± 6 days compared to 1.8 ± 2 days in Group II (*p* < .005). Forty-three Group II patients had non abdominal pathology (orthopedic, neurosurgical or urologic) that required admission for more than 24 h.

The presence of macrohematuria correlated with a change in management based on CT findings (*p* < 0.05). All 5 patients with macrohematuria were admitted. Four of them had renal injury and 1 patient had a liver laceration and pelvic fractures.

**Fig. 1:**
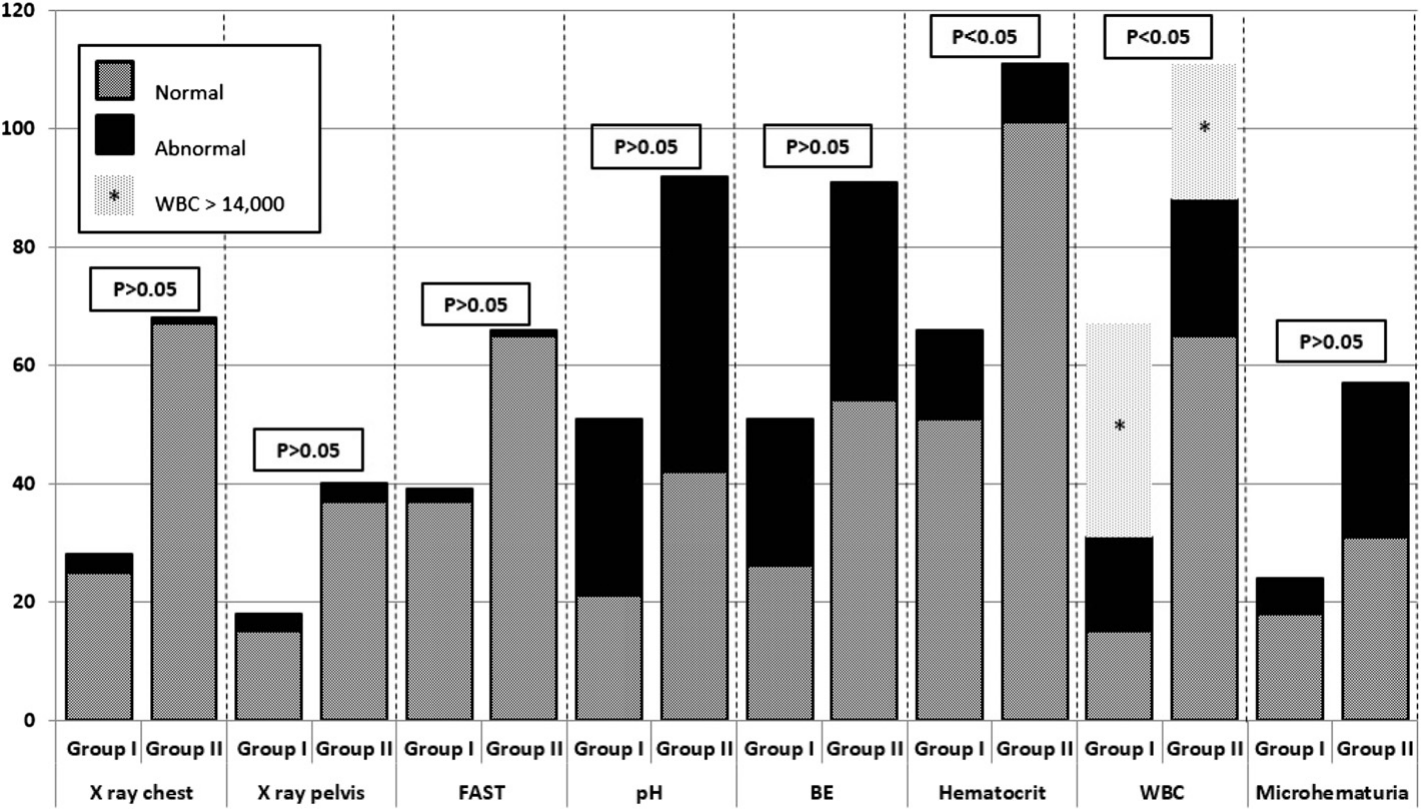
Laboratory tests and imaging study results

Laboratory tests were performed according to the decision of the surgeon on call, thus some tests results are missing in some of the patients. Detailed data is described in Fig. 1.

Markedly elevated WBC (>14000) and abnormally low hematocrit were significantly different between the groups (*p* < 0.05). Both correlated with abnormal CT findings requiring changes in patient management.

Abnormal pH, base excess and microhematuria did not predict positive CT findings (*p* > 0.05).

None of the initial imaging modalities (chest and pelvic X-rays, and FAST) were associated with intra-abdominal injury on CT.

## Discussion

The role of CT is well established in the management of adult blunt trauma patients [2]. Hemodynamically stable patients with an abnormal physical examination, patients with altered levels of consciousness and patients whose mechanism of injury suspicious for internal organ injury undergo further evaluation with CT [7]. However, in the pediatric population, recent studies have shown that using CT scan, based solely on the mechanism of injury, is not justified [3, 4].

The pediatric patient is considered 10 times more susceptible to radiation in terms of estimated cancer incidence [1]. There is an understandable tendency to define predictive rules for the identification of intra-abdominal injuries in order to limit the use of CT [8–15].

We continue to define the role of CT in the management of pediatric trauma patients. A liberal policy to perform abdominal CT in every case of suspected abdominal trauma has been changed to a more conservative approach in our Institution. The goal of this study was to define readily available clinical parameters predicting a positive CT influencing treatment plan. We focused on physical examination, laboratory tests and initial imaging findings. The mechanism of injury was already evaluated in our previous work [4], where it was singled out in order to define its sole role in prediction of abdominal CT findings. Therefore it was not used in the current study as a variable, but rather as a patient characteristic in the two study groups.

Despite some data supporting the association of positive physical findings with intra-abdominal injury [8–10, 12, 13], our work has shown no such relation. Because of the small numbers in our study, analysis of the positive physical findings by regions was found statistically insignificant. There was an inconsistency in the documentation of the physical examination, nevertheless it seems logical to assume that at least positive findings would have been documented. Though in other works [8–10, 12, 16] positive findings on the physical examination of abdomen, chest and long bones fractures were predictive of intra-abdominal injury, our results show a lack of influence of the physical findings on the chance to discover traumatic pathology on the CT.

None of the initial imaging modalities (chest and pelvic X-rays and FAST) were associated with intra-abdominal injury on CT in our study. Beck [17] has reported that an abnormal pelvic roentgenogram predicts a positive CT scan. However, Kessel et al. [18] have questioned the usefulness of routine pelvic x-ray in stable multiple trauma patients, as in patients undergoing CT it did not change the therapeutic policy [18]. Streck et al. [12] found an abnormal chest X-ray to be associated with intra-abdominal injury, but the pelvic or femur roentgenogramm bore no such association [12].

The use of FAST in the management of pediatric trauma patients is debated in the literature. Soundappan et al. [19] and Soudack et al. [20] reported high sensitivity of the test (81 % and 92.5 % accordingly). However, Scaife et al. [21] found the sensitivity to be 50 %, which seems unacceptably low. It was concluded in this study that true positive FAST exams are uncommon and would rarely direct management. The limited role of FAST in stable pediatric patients has been shown by other authors [22, 23].

In our study, 105 patients underwent FAST (Table 2). Three (2.9 %) were positive, and of them 2 had findings on the following CT that led to a change in treatment plan.

In general, there is a low prevalence of laboratory test abnormalities in moderately injured pediatric patients (5.7 %) [24]. Rovalis et al. [25] showed that patients with a severe head injury had significantly higher WBC counts [25]. Schnüriger et al. [26] evaluated the relationship between a hollow viscus injury and WBC counts and concluded that the diagnostic value of serial WBC tests in predicting a hollow viscus injury is very limited. Holmes et al. [8] evaluated clinical and laboratory parameters for the identification of intra-abdominal injuries in pediatric blunt trauma patients. They have found the WBC to be higher in patients with intra-abdominal injuries, but the authors could not identify a clinically useful cutoff that would distinguish between the groups. In our study, a WBC > 14000 strongly correlated with positive abdominal CT findings.

The utility of the hematocrit for screening for unsuspected IAI remains unproven [8]. We found that a low hematocrit level is a significant predicting factor for IAI. Base excess is considered a predictor of mortality and severity of injury in trauma patients. In pediatric trauma patients though, it has been shown to be a weak prognostic factor [27]. In our study, a negative base excess level was not associated with abnormal CT findings.

The presence of macrohematuria is invariably associated with intra-abdominal injury in children [8, 9, 11–13, 24]. In this respect, our study does not differ from previous studies.

The data concerning free peritoneal fluid in pediatric blunt trauma is inconsistent. Holmes et al. [28] found isolated intraperitoneal fluid in 8 % of pediatric blunt trauma patients undergoing abdominal CT, and IAIs subsequently identified in 17 % of these patients. The likelihood of intra-abdominal injury increased with increasing amounts of fluid. In another study, 16 % of patients with intra-abdominal free fluid required laparotomy [29]. On the other hand, Christiano found free intra-abdominal fluid on 14 % of CTs performed for pediatric blunt abdominal injury [30], but only 3 % of them developed peritonitis and required surgery. Although the majority of patients will be eventually discharged without any intervention, the amount of patients requiring is not negligible, and therefore careful follow up with serial examination is necessary in selected cases. In our study, free intraperitoneal fluid was found in 38 (20.8 %) patients without any identifiable cause on CT. Three of them (8 %) eventually required surgery. Two had more than a minimal amount of fluid on the CT, and 1 patient developed signs of peritoneal irritation during the observation. Small bowel and mesentery laceration were found on laparotomy.

Our institutional policy dictates overnight observation for every pediatric patient who has undergone an abdominal CT, even if it was normal. One of the reasons is the known propensity of the CT to miss hollow viscus and pancreatic injuries [13].

The major limitations of this study include its retrospective nature and the relatively small amount of patients. Many of the patients did not have all the examinations because they were probably deemed unnecessary at the time of evaluation. Prospective studies including a large number of patients are needed for further evaluation of the role of CT in the pediatric trauma population.

## Conclusion

Our data shows that an elevated WBC, a decreased hematocrit level and the presence of macrohematuria strongly implies abdominal CT findings leading to changes in management. The main influence resulting from CT was a change in the level of care (prolonged observation). It may also guide the need for active intervention in selected patients.

### Study presentation

This study was presented at the “Best of the Best” session during the Third World Society of Emergency Surgery Congress (WSES) that took place in Jerusalem, Israel in July 2015.
